# Rhizospheric Bacterial Community of Endemic *Rhododendron arboreum* Sm. Ssp. delavayi along Eastern Himalayan Slope in Tawang

**DOI:** 10.3389/fpls.2016.01345

**Published:** 2016-09-02

**Authors:** Rajal Debnath, Archana Yadav, Vijai K. Gupta, Bhim P. Singh, Pratap J. Handique, Ratul Saikia

**Affiliations:** ^1^Microbial Biotechnology Group, Biological Science and Technology Division, Council of Scientific and Industrial Research-North East Institute of Science and TechnologyJorhat, India; ^2^Molecular Glycobiotechnology Group, Discipline of Biochemistry, National University of Ireland GalwayGalway, Ireland; ^3^Molecular Microbiology and Systematics Laboratory, Department of Biotechnology, Mizoram UniversityAizawl, India; ^4^Department of Biotechnology, Gauhati UniversityGuwahati, India

**Keywords:** *Rhododendron arboreum* Eastern Himalaya, bacterial diversity, QIIME, UPARSE, *Acidobacteria*

## Abstract

Information on rhizosphere microbiome of endemic plants from high mountain ecosystems against those of cultivated plantations is inadequate. Comparative bacterial profiles of endemic medicinal plant *Rhododendron arboreum* Sm. subsp. delavayi rhizosphere pertaining to four altitudinal zonation Pankang Thang (PTSO), Nagula, Y-junction and Bum La (Indo-China border; in triplicates each) along cold adapted Eastern slope of Himalayan Tawang region, India is described here. Significant differences in DGGE profile between below ground bulk vs. rhizospheric community profile associated with the plant was identified. Tagged 16S amplicon sequencing from PTSO (3912 m) to Bum La (4509 m), revealed that soil pH, total nitrogen (TN), organic matter (OM) significantly influenced the underlying bacterial community structure at different altitudes. The relative abundance of *Acidobacteria* was inversely related to pH, as opposed to TN which was positively correlated to *Acidobacteria* and *Proteobacteria* abundance. TN was also the significant predictor for less abundant taxonomic groups *Chloroflexi*, *Gemmatimonadetes*, and *Nitrospirae*. Bum La soil harbored less bacterial diversity compared to other sites at lower altitudes. The most abundant phyla at 3% genetic difference were *Acidobacteria, Actinobacteria*, and *Proteobacteria* amongst others. Analysis of similarity indicated greater similarity within lower altitudinal than higher altitudinal group (ANOSIM, *R* = 0.287, *p* = 0.02). Constraining the ordination with the edaphic factor explained 83.13% of variation. Unique phylotypes of *Bradyrhizobium* and uncultured *Rhizobiales* were found in significant proportions at the four regions. With over 1% relative abundance *Actinobacteria* (42.6%), *Acidobacteria* (24.02%), *Proteobacteria* (16.00%), AD3 (9.23%), WPS-2 (5.1%), and *Chloroflexi* (1.48%) dominated the core microbiome.

## Introduction

The plant microbiome refers to the composite community of microbes associated with plants (Mendes et al., 2013). Plants constantly feed the adjacent rhizosphere, phyllosphere, endosphere, and spermosphere by depositing assimilated carbon thereby modulating microbial composition and activities. Complex communities of plant-associated microbes are an untapped reservoir that promotes plant health and productivity (Bisseling et al., 2009). By providing rhizodeposits viz; exudates, mucilage, border cells, the host plants diversify the members of bacteria, fungi, oomycetes, nematodes, protozoa, algae, viruses, archaea, and arthropods in the rhizosphere (Cook et al., 1995; Pierret et al., 2007; Raaijmakers et al., 2009). Moreover, rhizosphere specific differential selection of bacterial communities is displayed by different pioneer plant species: *Festuca halleri* All, *Gnaphalium supinum* (L.), *Leucanthemopsis alpina* (L.), *Linaria alpina* (L.) Mill, *Minuartia sedoides* (L.) Hiern, *Potentilla aurea* (L.), *Saxifraga bryoides* (L.), *Sedum alpestre* Vill., *Senecio carniolicus* (Willd.) Braun-Blanq, *Sibbaldia procumbens* (L.), *Silene acaulis* (L.) Jacq, and *Veronica bellidioides* (L.) in high mountain ecosystem and well documented (Ciccazzo et al., 2014).

Rhizospheric microbial communities are pivotal to natural ecosystems, maintenance of ecosystem diversity and in restoration projects through means of transplantation practices. Such practices apart from repairing landscapes also act as conservation tool for relocating plant communities, saving species from condemned locations, enhancing dwindling populations or creating new ones (Fahselt, 2007). Nevertheless, microbial communities are poorly understood and ignored while considering such practices. Lau and Lennon (2011) have shown that below ground rhizospheric microbial community has the power of modulating plant evolutionary process, thereby affecting patterns of natural selection on plant traits. By altering composition of below-ground microbial community with treatments, the authors showed *Brassica rapa* plants associated with simplified, less diverse microbial community compared to plants with highly diverse complex microbial community, were smaller, had reduced chlorophyll content, produced less flower and had less fecundity. Such reports relate to the reciprocal interactions that exist between the plant and associated microbes in modulating each other. With a significant fraction of global plant taxas classified as endangered, endemic, threatened or rare, long term sustainability and conservation strategies involving transplantation practices may be strengthened with an in-depth understanding of the plant microbiome as outlined and exemplified by global organizations. Fate of rare, threatened or endangered plants is often tied to undisturbed habitat, intact communities and relocation difficulties arise in moving such single species (Fahselt, 2007). Reports on the rhizosphere microbes are concentrated mostly on the complex mycorrhizal communities in the domesticated plants from soil samples to which these plants were once introduced. Shen and Wang (2011) showed that transplanted seedlings of endemic and critically endangered *Euryodendron excelsum* with native arbuscular mycorrhizal fungi enhanced survivability by 80% compared to 46% on their own. Such studies suggest that below ground microbial communities may play important role in determining diversity, productivity and composition of above ground plant community. Alternatively, phylochip, pyrosequencing approaches are limited to maize crop, oat in microcosm/forest soil, sugar beet, potato, *Arabidopsis thaliana* to mention a few (Mendes et al., 2013). Often overlooked, microbiome studies of endemic medicinal plants are still primitive.

The Eastern Himalayan (EH) biodiversity hotspots (an amalgamation of Indo Malayan, Palaearctic and Sino-Japanese region) covers an area of 5,25,000 km^2^, spreads from the Kaligandaki Valley, Kosi Basin, Mechi Basin (central Nepal) to Yunnan province (northwest China) (Shrestha et al., 2010) and incubates 200 Global ecoregions (Sharma et al., 2010). Of the Global Biodiversity Hotspots (34) EH encompasses 25 ecoregions, of which 19 have high conservation significance in terms of their global conservation value (Shrestha et al., 2010). Myers et al. (2000) predicted that the Indo-Burma Hotspot alone shelters 2.3% of global endemic plants and 1.9% of global endemic vertebrates with a species density of 7.0 and 0.5 per 100 km^2^ area, respectively.

The Tawang district (27.58 N 91.86 E, elevation 3048 m) in Arunachal Pradesh (hereafter AP) falls in the alpine shrub bioclimatic zone, experiences a high number of frost/ice days and high precipitation. Toward the extreme north in Pankang Thang, Nagula mostly conifer trees, rhododendron shrubs, alpine meadows are prevalent. The vegetation above the tree line entertains a community of dense juniper, *Ponerorchis* spp., *Boschniakia* spp., *Saussurea obvallata* and *Rhododendron* shrubberies that extend to about 4,500 m (Sekar and Srivastava, 2010). Many high altitude medicinal plants such as *Fritillaria cirrhosa*, *Aconitum*, and Rheum are also found in this region. *Rhododendron* genera are widely distributed at higher elevations in the Sino-Himalayan regions. A maximum concentration of 86% species is observed in AP. Pharmacologically, anti-inflammatory, anti-nociceptive, anti-diabetic, hepatoprotective, antioxidant, and anti-diarrhoeal activity of *Rhododendron arboreum* is reported in potential animal models (Hariharan and Rangaswami, 1966; Swaroop et al., 2005; Verma et al., 2011; Srivastava, 2012).

Although, EH is documented as biodiversity hotspot, microbial communities of cold adapted endemic plants of this region was not previously assessed. Such mountain ecosystems are under constant pressure of loss in biological diversity and are vulnerable due to climate change. Such issues are addressed by the special programs under the Convention on Biological Diversity (CBD) which aims at reducing the loss at global, regional, or national levels in the EH region.

The aim of the present work was to assess the colonization pattern of the rhizospheric bacteria in medicinally important endemic *R. arboreum* Sm. Subsp. delavayi occurring at high altitude acidic soils using Illumina based 16S sequencing. Plant specific selection of rhizospheric bacterial community is reported for field grown agricultural plants (Smalla et al., 2001) and different pioneer plants in high mountain ecosystem (Ciccazzo et al., 2014) but variable results were reported in non-agricultural perennial bunch grasses, herbaceous plants. We used DGGE fingerprints to describe whether the plant species effect on rhizospheric bacterial community composition exists in endemic medicinal *R. arboreum* plant in natural high mountain ecosystem along altitudinal blocks. To find out any influence of edaphic factors, elevational gradients variables were scaled on to dominating groups, sub-groups that were identified by amplicon sequencing. Efforts in understanding the rhizosphere of medicinal, endemic plant species will be vital for bioprospection of ecto- and endophytic microbes and in selecting suitable receptor sites for *ex situ* conservation, transplantation models.

## Materials and Methods

### Sampling Site Description

The samples collected along an increasing altitudinal gradient from four geographical regions with three replicates each from [PTSO (Pankang Thang, 17 km upstream of Tawang town at around 3912 m; P.B.4, P.C.8, P.E.1); Nagula (around 3 km upstream of PTSO at 4140 m; N.D.1, N.4.8, N.1.4); Y-junction (at an altitude of ∼4400 m and 4 km upstream of Nagula; Y.9.1, Y.3.8, Y.5.4); Bum la (37 km upstream of Tawang town at 4509 m; B.6.8, B.7.4, B.10.1)] were mostly acidic loamy tundra soil. Short statured actively growing plants of roughly the same developmental stage (flowering) with associated soils were collected after removal of subsurface litter from each altitudinal site within each region type during the month of October 2013. A trenching shovel was used to expose the rocky coarse surface and recovery push probe (1/2 inch to 40 inch) to reach the deeply rooted zone and 0.5–1.0 kg root-adhered composite soil (without compromising the integrity of the plant) was collected and aseptically transferred into separate sterile polythene bags [Hi-dispo Bags (Himedia)] and transported to the laboratory by chilling on ice. The root plus loosely adhered soil was separated from the bulk of the soil. Weakly adhered soil was discarded by method of gentle shaking, exposing the roots. Soil firmly associated with the root surface was scraped off, sieved through 2.0 mm screen to remove rootlets and other significant particles. One portion of composite soil was kept in 50 ml centrifuge tubes at -20°C freezer until molecular analysis.

Soil physicochemical properties were determined by standard reported methods (Gee and Or, 2002). Briefly, soil gravimetric moisture content (MC) was measured by difference in weight between moist samples as collected from the field and corresponding dried samples (100°C for 48 h). Chemical analysis of soil samples was done after acid digestion using Atomic absorption spectrophotometer (Perkin Elmer AAnalyst 800) and standard procedure such as: inorganic nitrogen (NH_4_^+^ and NO_3_^-^) extracted using a 2 M KCl solution and filtered using Whatman No. 42 paper. Plant available phosphorous (P) in a Bray extract reacting with ammonium molybdate, pH of the soil in 1:10 dilution (soil to water, w/v; Fixen and Grove, 1990); exchangeable cations were extracted using 1 mol L^-1^ neutral ammonium acetate solution and quantified through the Kjeldahl method to measure the cation exchange capacity (CEC; Chapman, 1965), Walkley-Black method for organic matter determination (SOM; Nelson and Sommers, 1982) and total carbon (TC), nitrogen (TN) measured by combustion at 950°C in an elemental analyzer. All quantified soil properties are given in Supplementary Table S1.

### Soil DNA Purification and Real Time qPCR Assay

Soil genomic DNA was extracted from ∼500 mg of soil using PowerSoil^®^ DNA isolation kit (Mo Bio Laboratories, Inc.) by method of bead beating and spin column purification using manufacturer’s protocol. OneStep^TM^ PCR Inhibitor Removal Kit (Zymo research) was used for DNA isolation with high humic acid content to overcome PCR failure. The isolated DNA was checked for purity and integrity in 0.8% agarose gel. DNA quantification was done using BioSpectrometer (Eppendorf) and stored at -70°C. Quantification of total bacteria, group specific *Actinobacteria, Acidobacteria*, α-*Proteobacteria*, and β-*Proteobacteria* was conducted on a StepOnePlus^TM^ qPCR thermocycler with Power SYBR green PCR master mix (Applied Biosystems Pvt. Ltd.). ROX dye acted as internal passive reference to normalize non-PCR related fluorescence fluctuations. Combination of primers (Eub338, Eub518, Actino235, Acid31, Alf685, and Bet680) and conditions used in the set-up were described previously (Fierer et al., 2005). Known template standards were prepared from bacterial clones using primers as described previously (Navarrete et al., 2013), (PureLink PCR purification kit; Thermo fisher Scientific) ligated into pGEM-T easy vector and transformed into Escherichia coli Dh5α-competent cells. On confirmation of positive transformants using primer pair SP6/T7, plasmid DNA was isolated (PureLink Quick plasmid Miniprep kit; Thermofisher) and integrity checked on 1% agarose gel. Standard curves were generated by triplicates of 10-fold dilution series ranging 1.75 × 10^-2^ to 1.75 × 10^-7^ ng μL^-1^ of DNA per reaction. Target copy numbers of taxonomic groups in metagenomic samples were obtained from the curve. One-way ANOVA followed *post hoc* analysis using Tukey’s HSD test was used to determine the significance of the differences between rhizospheric soil samples.

### DGGE Analysis of Bacterial Community from Total Soil DNA

16S-rDNA component of the total soil DNA from bulk and rhizospheric samples was amplified using V3-GC clamped primer pairs as described earlier (Muyzer et al., 1993). The products were analyzed by DGGE fingerprints by resolution in 8% gradient polyacrylamide gel (40–60% denaturant) and image recorded for processing and downstream analysis. The fingerprints were evaluated using Gelanalyzer software 2010. Normalization was done by subtracting background from the profiles by employing automatic rolling ball method. All fragment data from multiple samples were combined and sorted by physical distance in the gel and clustering was done in a two step manner as described earlier by Ishii et al. (2009). In the first step the fragments from different samples having identical values were clustered and sorted together. In a second step clustering was done if the physical distance is smaller than the cutoff value and originating from different samples. The resultant output of matrix so obtained by clustering is used to generate a dendrogram using the “pvclust” package in R statistical language. The probability values for each cluster were obtained by multiple bootstrap sampling. Permutation tests for the comparison of groups of lanes (bulk from rhizosphere) were based on Pearson’s product moment correlation (Kropf et al., 2004).

### V3 Amplification, Library Generation, Illumina Sequencing

To evaluate the diversity as well as composition of bacterial communities’ protocol described by Caporaso et al. (2012) was followed. Three extraction of DNA from each sample/site were pooled into one and sent for V3 sequencing to Scigenom Pvt. Labs, Cochin, India on Illumiina Miseq platform. Amplification was carried out in two steps to create a 150 bp (x2) paired end sequencing library. 16S rDNA V3 targeted loci amplification was done using primer pair 341F: 5′-CCTACGGGAGGCAGCAG-3′ and 518R 5′-ATTACCGCGGCTGCTGG-3′ for construction of community library. PCR cocktail in triplicate contained 2 μL of each 10 pmol μL^-1^ forward and reverse primers, 0.5 μL of 40 mM dNTP, 5 μL of 5X Phusion HF reaction buffer, 0.2 μL of 2 U μL^-1^ F-540 Special Phusion HS DNA Polymerase, 5 ng input DNA and water to make up the total volume to 25 μL. Cycling condition consisted of: initial denaturation at 98°C for 30 s, 35 cycles of 98°C for 10 s (denaturation), 72°C for 30 s, extension at 72°C for 5 s followed by holding at 4°C. Size selected gel purified amplicons of 230–250 bp were utilized for a second PCR as template. Second PCR contained 50 μL volumes with same master mix proportion but with Illumina Truseq adapters and indexes. This generated library is of 354–362 bp. The raw fastq files and associated metadata were submitted to the NCBI Short read archive under **SRA study ID**: **SRP049018**.

### Sequence Processing and OTU Picking

Sequences were pre-processed and analyzed using QIIME (Quantitative Insights into Microbial Ecology; Caporaso et al., 2010b) and UPARSE pipeline (Edgar, 2013). The merged pairs were quality trimmed and filtered with varied maximum expected error of 0.25 and 0.5 and 1.0 (Edgar and Flyvbjerg, 2015). Sample pooling and global trimming was applied prior to further processing. OTUs were clustered on de-replicated and singleton removed files at an identity of 97% using usearch v7. Reference based chimera filtering was done against greengenes and GOLD database using Uchime (Edgar et al., 2011). The high quality centroid sequences (<1% incorrect bases) were taxonomically assigned with the assign_taxonomy.py command in QIIME against the greengenes database v13_8 (DeSantis et al., 2006) at default settings with RDP classifier (Wang et al., 2007) against 80% confidence level threshold. Representative sequences were PyNAST multiple aligned using align_seqs.py and filtered for only informative sites (Caporaso et al., 2010a) using infernal (Nawrocki et al., 2009) aligner. Fasttree v.2.1 (Price et al., 2010) was implemented in building the approximately maximum-likelihood phylogenetic tree for β-diversity studies using UniFrac metric. Phyloseq package (McMurdie and Holmes, 2013) was used for detailed downstream analysis on the abundance matrix rarefied to an even depth with 10 iterations to cancel out likely bias on diversity calculation due to uneven sequencing depth. Additional sequence error filtering was performed to remove spurious OTU’s appearing in fewer than half of the samples and containing fewer than five reads to reduce the PCR and sequencing based bias, following which the table was again rarefied as above. With representative sequences from the 10 most abundant OTU’s (extracted using Biostrings package) across all samples, we used Eztaxon-e interface to find the best high-quality matches ≥ 1200 bp in the curated RDP SSU database.

### Diversity and Statistical Analysis

Differences in community richness or relative abundance of individual bacterial taxa’s were tested for significance using non-parametric Kruskal–Wallis test after bonferroni correction was carried out. Significance was determined by *p*-value at an α of 0.05 calculated from default number of Monte Carlo permutations (999) for the non-parametric tests. To compare community composition between sample types, we used vegan to compute a Bray–Curtis dissimilarity matrix after Hellinger transformation of the count data. Comparison between bacterial community structure and different groupings were tested using Analysis of similarity (ANOSIM; permutations 999) together with non-metric scaling. The phylogenetic metric Unifrac (Lozupone and Knight, 2005) was used to calculate β-diversity within and between the samples. A Mantel test on distance dataset was done to determine whether soil physical and chemical properties, if any, were significantly correlated with the bacterial community. Constrained ordination of soil parameters and community composition was carried out using capscale function and envifit was used to partial out factors not contributing significantly to the ordination. Core microbiome analysis was done using the compute_core_microbiome.py command in QIIME with stringent parameter of 100% sample frequency.

## Results

### Analysis and Comparison of DGGE Community Profiles

**FIGURE 1:**
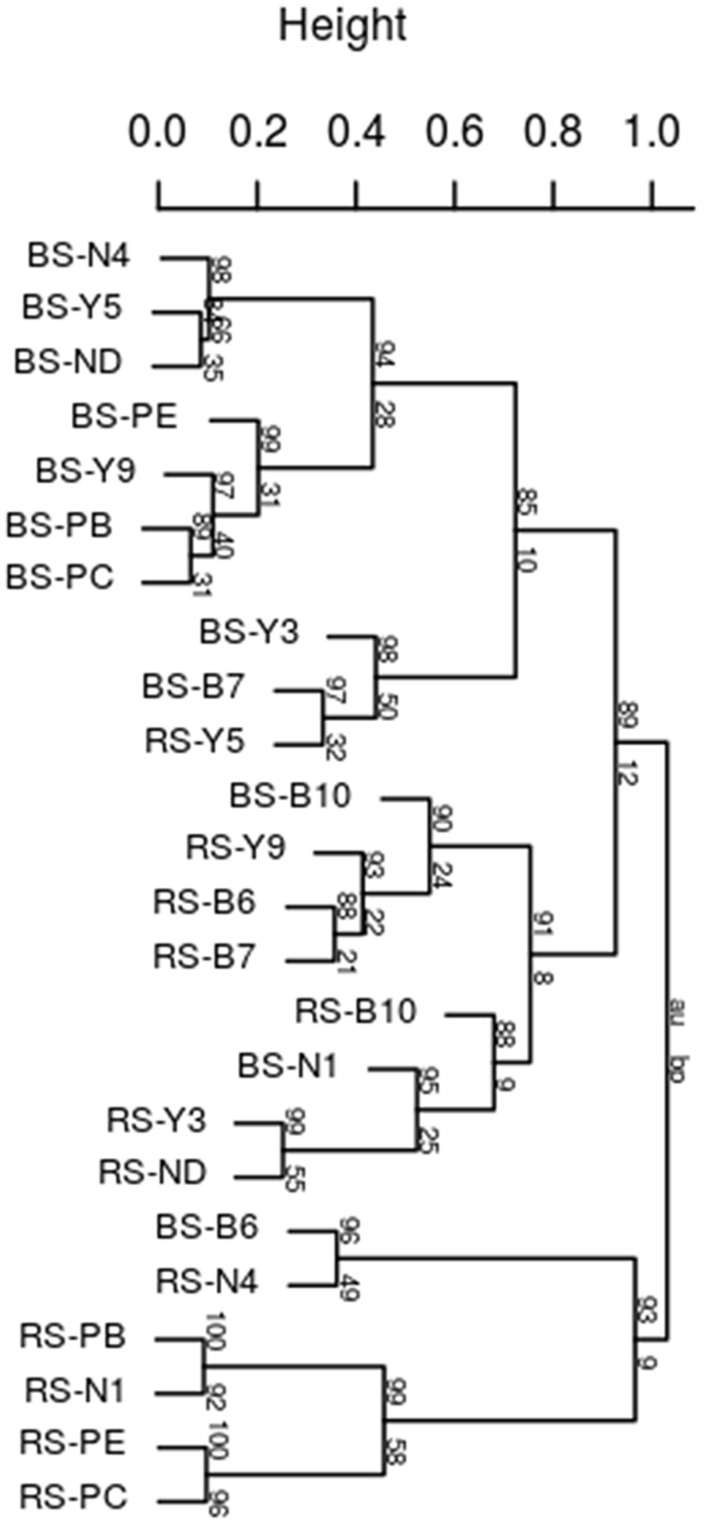
**Dendrogram of bacterial community fingerprints**. UPGMA cluster analysis based on Pearson correlation distance obtained by DGGE samples taken from bulk soils (b) and the corresponding *Rhododendron arboreum* rhizosphere. Approximately unbiased (au) *p*-values at the edges of the cluster were obtained by multiple bootstrap resampling (*n* = 1000).

*Rhododendron arboretum* rhizospheric soil fingerprint was markedly having distinct profiles and higher intensity bands compared to the bulk soil 16S fragments (Supplementary Figure S1) indicating enrichment of specific taxonomic groups in the rhizosphere. Significant differences between bulk soil and rhizospheric soil within each altitudinal site was observed indicating significant rhizospheric effect (*R* = 0.3033, *p* = 0.002). In addition, when the combined bulk community profiles from four altitudinal blocks were compared to combined profile of rhizospheric community, significant differences was observed (*R* = 0.3294, *p* = 0.005) indicating strong rhizosphere mediated selection of community despite geographical separation. This was confirmed with the cluster analysis based on Pearson correlation coefficients that grouped bulk from rhizospheric soil with strong bootstrap value (**Figure 1**). A strong shift in the rhizospheric community pattern of *R. arboreum* along the altitudinal gradient can be observed for RS-N1 (Nagula) grouped within PTSO (RS-PB, RS-PE, and RS-PC) at a lower altitude and also for RS-Y9 (Y-junction) clustered together with rhizospheric Bum La soil (RS-B6, RS-B7, and RS-B10) at a higher altitude. This may be due to the relatively short interval between PTSO-Nagula sampling locations.

### Bacterial Gene Abundance and Diversity Estimates

The distinct community pattern of rhizospheric soil than bulk soil revealed by DGGE was further calculated for diversity indices as measure of community complexity. The sequence datasets obtained from rhizospheric soil were normalized respective to the lowest minimum number of reads (358728) to account for bias in diversity overestimation. The rarefied data (Supplementary Figure S2) showed significant difference (*p* = 0.02) between the sample groups in terms of richness estimates. Following *post hoc* test showed two distinct categories, PTSO (*p* = 0.014) and Nagula (*p* = 0.007) were significantly different from Bum La soil, however, Bum La was not significantly different from Y-junction site (**Table 1**). Y-junction and Bum La soil rhizosphere harbored comparatively less diversity, whereby Nagula and PTSO soil represented the highest diversity.

**Table 1:** Non-parametric Kruskal–Wallis test with monte carlo permutations (999) to test significant differences (*p* ≤ 0.05) in α-diversity measures among the lower and higher altitude groups of rhizospheric community.

	Observed		Chao1		ACE		Inv-Simpson		Fisher	
	Low	High	Low	High	Low	High	Low	High	Low	High
Minimum	2794	2352	3012.1	2596.1	2987.8	2576.7	98.8	51.6	413.2	337.8
Maximum	4120	3145	4329.1	3317.2	4284.4	3301.7	306.4	223	653.6	474.9
Mean	3213.5	2675.33	3422.48	2867.71	3396.45	2855.25	183.48	136.2	488.2	393.15
Standard deviation	478.4	283.57	481.07	275.23	469.7	277.29	79.19	74.04	86.9	49.07
*p* = 0.05	0.0271		0.0256		0.0240		0.3886		0.0279	

*PTSO and Nagula rhizosphere were categorized as lower whereas Y-junction and Bumla as higher altitude*.

**FIGURE 2:**
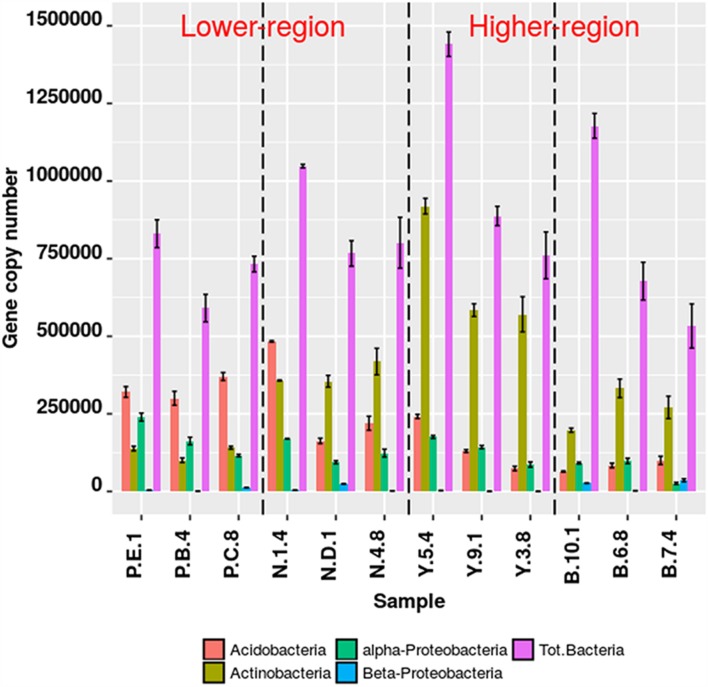
**Bacterial gene abundance (copy number) from rhizospheric soil at different altitudes**. Specific detection of bacterial groups by qPCR using taxon specific primers. Absolute gene copy numbers were determined from 5 ng of template DNA dilution curve series with known standards. Error bars are one standard error of the mean (SEM) of replicate qPCR reactions (*n* ≥ 3).

The qPCR assays for the target group *Actinobacteria*, *Acidobacteria*, α-*Proteobacteria*, β-*Proteobacteria* were 95.89% efficient (s.d. ± 7.80%). Significant differences in copy number abundances of rhizospheric *Actinobacteria* were observed between Y-junction and PTSO (*p* = 0.008), Nagula (*p* = 0.02), and Bum La (*p* = 0.0053). Acidobacteria abundances also varied between the lower altitudinal (PTSO) and higher altitudinal (Bum La) region (*p* = 0.02). qPCR results of pooled DNA extracts from replicate samples resulted in mean values within the deviation of the true replicate (**Figure 2**). Relative fractional abundances of the phylum (*Actinobacteria* and *Acidobacteria*) were not significantly different between the qPCR and Illumina sequencing approach suggesting unbiased target amplification.

### Taxonomic Composition at Different Sites

**FIGURE 3:**
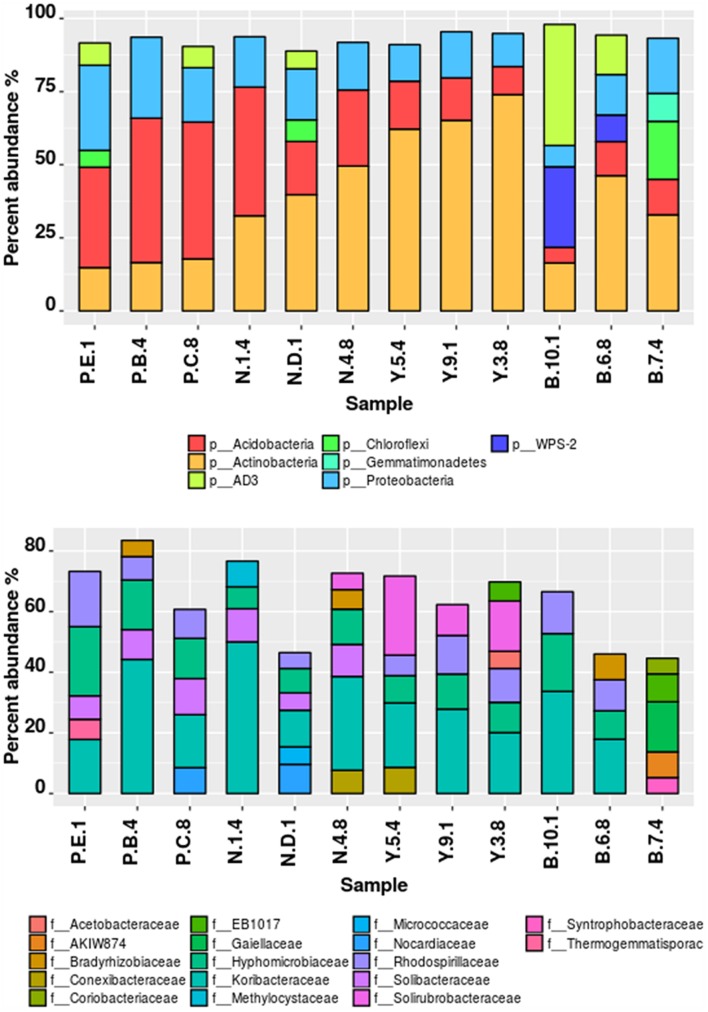
**Taxonomic composition of bacterial communities associated with *Rhododendron arboreum* rhizosphere sites along an altitudinal gradient with increasing pH**. Relative percentages of read abundance of different phylum and family level taxonomy within the different communities. **(A)** Sequences that could not be classified into any known phylum were removed. **(B)** At the family level differentiation only taxa with >1% abundance were included and not belonging to any known family were removed.

**FIGURE 4:**
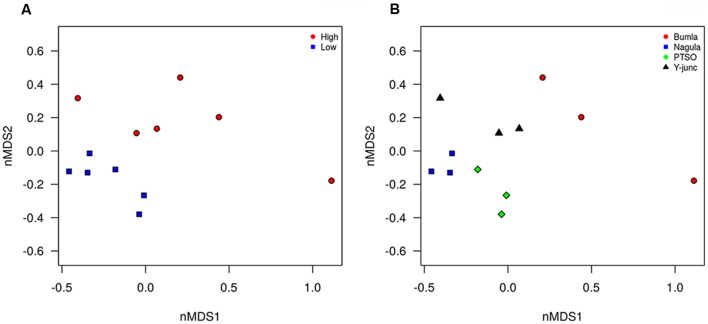
**Non-metric multidimensional analysis of bacterial community composition according to amplicon sequence data**. The relative abundance of OTU’s (Hellinger transformed) and dissimilarity based on Bray’s distances ordinated. **(A)** PTSO and Nagula (lower altitude) community more similar to each other than to Y-junction and Bum La (higher altitude) community. **(B)** Sample replicate within each region more similar that between sample replicates (*p* < 0.05).

Illumina sequencing provided insight into the taxonomic classification of rhizospheric soil community. In terms of community membership *Acidobacteria* (23.08%), *Actinobacteria* (40.57%), *Proteobacteria* (16.23%), *AD3* (8.24%), WPS-2 (5.5%) *Chloroflexi* (3.18%), *Firmicutes* (0.99%), *Gemmatimonadetes* (1.02%), and *Nitrospira* (0.47%) were present in all the four rhizospheric soils (**Figure 3**). *Acidobacteria* populations (*p* = 0.0008) were 3.14 times more in lower region than upper region. Also, *Proteobacteria* proportion (*p* = 0.021) of 13.3 vs. 21.3%, respectively, for upper and lower region rhizosphere were significantly different. In one of the Bum la soils (B.10.1), one OTU_6 solely accounted for 27.6% WPS-2 and OTU_5 accounted for 41.5% AD3 proportions. Non-metric plot (stress 0.02) showed greater community similarity across the high altitudinal soils than the lower altitudinal soil at PTSO and Nagula (*R* = 0.338, *P* = 0.02; ANOSIM; **Figure 4A**). Further within group community was found more similar than between group community (*R* = 0.3889, *p* = 0.002; **Figure 4B**) which may be accounted for the local microenvironment of the soil.

The represented sequences of the top 10 dominant OTU’s (over 1% abundance) in the entire dataset were also identified. Of the dominant ones OTU_8 with 99.3% identity with cultured isolate was identified as *Bradyrhizobium valentinum*, a novel bacterium isolated from root nodules of endemic plant *Lupinus mariae-josephi* (Sánchez-Cañizares et al., 2011). The rest of the sequences did not have any cultured counterpart as yet. The closest match for the rest of the dominant OTU’s with their respective detection source is mentioned in **Table 2**.

**Table 2:** List of top 10 dominant OTU’s (centroid sequences) accounted for over 1% total reads used to find the closest representative in RDP, SILVA, or NCBI through EZtaxon-e server.

OTU number	Taxonomic classification	EZTAXON-E closest match^a^	PS (%)	Hits isolation source^b^	PTSO (%)	Nagula (%)	Y-junction (%)	Bum La (%)
OTU_5087	o__Actinomycetales	Streptosporangiales; EU861937_s	97.35	Nitrogen fertilization on alpine tundra soil	0.5	3.112	5.9	1.024
OTU_5	p__AD3; c__ABS-6	Unclassified chloroflexi; AY913277_s	99.34	Duennwald forest, pH 3–5, humus 4–6%, C/N ratio 18–20	1.648	0.74	0.344	3.512
OTU_8	f__Bradyrhizobiaceae	*Bradyrhizobium valentinum*; JX514883	99.34	Root nodules of *Lupinus mariae-josephi* endemic plant	1.354	1.804	0.798	0.882
OTU_4	g__Rhodoplanes	Unclassified Rhizobiales; AB240329_s	99.34	Biofilm of Phragmite rhizosphere	2.439	1.045	0.81	0.473
OTU_5095	o__Actinomycetales	Unclassified_Actinomycetales; AY326625_s	98.01	Western Amazon forest soil in terra preta	0.104	0.121	3.959	0.123
OTU_4412	o__Actinomycetales	Unclassified Actinomycetales; EU861937_g	97.35	ND	0.206	1.202	2.215	0.319
OTU_6	p__WPS-2	Unclassified Clostridia; JN023717_s	99.34	Lichen and moss crust (temperate highland grassland)	0.61	0.401	0.476	2.01
OTU_13	p_AD-3; c__JG37-AG-4	Unclassified bacteria; FR749816_s	98.68	Antarctic Peninsula soil	1.029	0.198	0.057	1.639
OTU_3	p__Acidobacteria; f__Coriobacteriaceae	Acidobacteria_GP1; AY913298_s	99.34	Duennwald forest, pH 3–5, humus 4–6%, C/N ratio 18–20	0.789	1.198	0.692	0.219
OTU_10	o__Acidimicrobiales	Aciditerrimonas; PAC000213_s	99.34	ND	0.189	0.651	0.777	1.143

*^a^EZTAXON-E database (database of 16S rDNA sequences of validly described names of species with type strains and uncultured representatives, details of the database contents are available at http://www.ezbiocloud.net/eztaxon/database).*

*^b^Data for Hit isolation sources were from NCBI, SILVA browser*.

### Spatial Variation of Bacterial Community Structure

The association between the measured soil parameters and taxa profiles (*r* = 0.3512 and *p* = 0.005) from the four sites was confirmed by the Mantel correlation value. Soil pH was a major determining factor in shaping the overall changes in OTU distribution and was correlated with all the soil sites (*r* = 0.38 and *p* = 0.004). Besides pH, OM (*r* = 0.306, *p* = 0.029), TN (*r* = 0.2625, *p* = 0.035), NO_3_^+^ (*r* = 0.2131, *p* = 0.028), and NH_4_^+^ (*r* = 0.3809, *p* = 0.003) influence was also observed on the overall bacterial community composition. Different soil properties, however, influenced the two different groupings of soil sites. In the upper group (at relatively high elevation) only TN (*r* = 0.78, *p* = 0.018) and CEC (*r* = 0.81, *p* = 0.02) whereas the lower altitude soils was associated with Mg (*r* = 0.54, *p* = 0.05; **Table 3**).

**Table 3:** Mantel’s test statistic for significant correlation between soil physicochemical properties and bacterial communities.

Soil physical and chemical properties	All sample sites		High(Bum La and Y-junction)		Low(PTSO and Nagula)	
	*r*	*p*	*r*	*p*	*r*	*p*
Temp	0.222	0.103	0.203	0.78	0.0071	0.456
OM	0.306	**0.02^∗^**	0.1857	0.33	-0.192	0.74
pH	0.38	**0.004^∗∗^**	0.157	0.344	0.63	**0.005^∗∗^**
TN	0.2625	**0.043^∗^**	0.789	**0.018^∗^**	0.12	0.283
TC	-0.107	0.673	0.575	0.152	-0.0142	0.423
C:N	0.008	0.407	-0.3607	0.883	-0.246	0.82
P	-0.148	0.756	0.053	0.358	-0.227	0.819
Mg	0.2411	0.077	0.585	0.136	0.542	**0.05^∗^**
Moisture	0.1571	0.344	0.803	**0.02^∗^**	0.089	0.333
NO_3_^+^	0.213	**0.043^∗^**	-0.314	0.833	0.058	0.372
NH_4_^+^	0.38	**0.005^∗∗^**	0.157	0.344	0.007	0.456

*Datasets were permuted 999 times to obtain Spearman’s rank correlations (*r*) and significance (*p*). Temp, Temperature; OM, organic matter; TN, total nitrogen; TC, total carbon; C:N, ratio of carbon to nitrogen. Significant values ^∗^*P* < 0.05, ^∗∗^*P* < 0.005 are in bold*.

**FIGURE 5:**
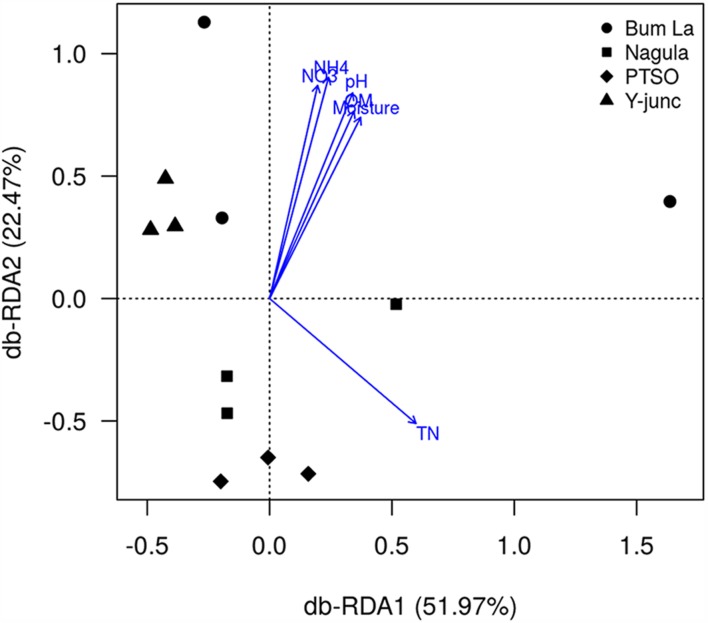
**Redundancy analysis of the effect of discriminative soil parameters on bacterial communities**. Numbers in parenthesis indicate the percentage of total variation explained by each axis. Only soil parameters which significantly (*p* < 0.05 by 1000 permutation tests) explained the bacterial community variation are shown.

The soil of PTSO region and Nagula had comparatively less pH which reflects the gradual decrease in relative abundance of phylum *Acidobacteria* (spearman’s rank correlation, *r* = -0.818, *p* = 0.002) as we go uphill toward Bumla region. In contrast, TN content (*r* = 0.629, *p* = 0.03) had an opposing effect on relative abundance of *Acidobacteria*. Actinobacterial populations were significantly high in Nagula and Y-junction region and none of the measured parameters could explain the changes in relative abundance values at different sites. Relative abundance of *Proteobacteria* changes was negatively correlated with NO_3_^+^ (*r* = -0.573, *p* = 0.05). Among the minor taxonomic groups relative abundance of *Chloroflexi* (*r* = 0.706, *p* = 0.01), *Nitrospirae* (*r* = 0.776, *p* = 0.004) and *Gemmatimonadetes* (*r* = 0.67, *p* = 0.01) was positively correlated with TN content. Phylogenetic diversity at four altitudinal sites had significantly different community profile, unweighted Unifrac (*p* = 0.042).

db-RDA could find that the fitted variables OM(*r* = 0.72, *p* = 0.003), pH (*r* = 0.84, *p* = 0.001), TN(*r* = 0.63, *p* = 0.003), MC (*r* = 0.70, *p* = 0.002), NO3 (*r* = 0.82, *p* = 0.001), and NH_4_^+^ (*r* = 0.90, *p* = 0.001) had significant relationships with the first two PCs. pH (MC, OM, NH_4_^+^, NO_3_^+^ positively collinear) and TN were the main factors influencing the bacterial community composition (**Figure 5**). In total 74.44% of the variation in the bacterial community could be significantly explained by TN and pH of the soil. Discriminative parameters other than pH and TN explained some amount of variation within the sub-community of *Proteobacteria* (46.18% by Mg and TN, Supplementary Figure S3), *AD3* (42.95% explained by Mg content, Supplementary Figure S4), *WPS-2* [52.94% by Mg and NH_4_^+^ content (OM, NO_3_^+^, MC collinear with the vector), Supplementary Figure S5], *Chloroflexi* (50.77% by MC, Supplementary Figure S6), *Gemmatimonadetes* (51.29% explained by TN and MC, Supplementary Figure S8), *Nitrospirae* (59.95% by MC and Mg content, Supplementary Figure S9). None of the measured parameters, however, could explain the variation in *Firmicutes* community (Supplementary Figure S7).

Shared phylotypes among the four sites shows an overlap of 300 OTU’s (61.1%) in the core microbiome (Supplementary Figure S10). The *Actinobacteria* population increased from the core to the variable microbiome while *Proteobacteria* decreased. Ninety five of these shared OTU’s were >0.1% abundant and 20 OTU’s were >1% abundant of the total microbiome.

## Discussion

We assessed the rhizospheric bacterial community of endemic medicinal plant *R. arboreum* Sm. subsp. delavayi and its association with soil properties at a high altitude region upstream of Tawang, India situated in the eastern slope of the Himalaya. DGGE profiles of bulk soil and rhizospheric soil ruled out the possibility for specific bacterial group selection by the dry, cold Himalayan soils and emphasized on the phenomenon of “rhizosphere effect.” In order to understand the mechanisms involved in specificity (adherence, recognition) and rhizosphere enrichment processes, microbial ecology studies of rhizosphere from native plants in natural ecosystem other than agricultural or model crops will be important. Comparative, biogeographic studies will help in assessing the possible role of rhizospheric microbial communities as selective constraints for the diversification of more than 420000 plants (Berg and Smalla, 2009). Many previous studies on rhizosphere bacterial community have identified soil type and plant specificity as one of the major factor in shaping microbial communities. Significant differences among the community composition based on both DGGE fingerprint and pyrosequencing sequencing analysis of 16SrRNA amplicons from bulk and rhizosphere soil of lettuce (cv. “Tizian”) grown in three soils diluvial, alluvial and lowess was identified (Schreiter et al., 2014). In addition a strong partitioning effect between bulk and rhizospheric soil of lettuce was also observed at two different time points (3 and 7 weeks post planting). Similarly differing relationship between C concentrations (and in some experiments N) along the root and the distribution and abundance of oligotrophic and copiotrophic bacteria in the bulk and rhizospheres of lettuce and tomato was reported by Maloney et al. (1997). Soil type dependent composition of bacterial communities in the rhizosphere of *A. thaliana* grown under greenhouse conditions in different soils was also observed (Bulgarelli et al., 2012; Lundberg et al., 2012). Although the same phyla were reported in the rhizosphere of different plants viz; oak, *A. thaliana*, Bt- and conventional maize variety (Uroz et al., 2010; Bulgarelli et al., 2012; Lundberg et al., 2012; Dohrmann et al., 2013) their relative abundances substantially differed to that of bulk soil.

We observed a significant difference in bacterial community structure at different altitudes but could not find a difference in diversity values along the altitudinal trend, as opposed to other studies supporting the Baas-becking hypothesis (Finkel et al., 2012). This might be due to significant intervals between sampling sites and requires considerably large sampling effort at short incremental intervals to comment on it. In another study pertaining to the biogeographic distribution of soil archaeal and bacterial communities across three vertical climate zones (3,106–4,479 m.a.s.l.) in Mt. Shegyla using pyrosequencing approaches the authors showed altitudinal distribution patterns of soil bacterial and archaeal assemblages with increase in elevation (Wang et al., 2014). Similar altitudinal trends were found in Northern Slope of Changbai Mountain, China (Zhang et al., 2013). According to another study in the Colorado Rocky Mountains, phylogenetic β-diversity of bacterial lineages were not randomly distributed, but rather had a gradient dependent spatial structure (Bryant et al., 2008).

The Mantel’s statistic identified pH, OM and TN in the soil were reshaping the community composition differences as observed by Illumina sequencing. The pattern of specific bacterial phylum responses to pH gradients is well documented (Lauber et al., 2009). In a gradient dependent study comprising soil bacterial communities across six elevations with vegetation types ranging from forest to alpine tundra in Mt. Changbai, China, pH was established to be the best predictor for community composition (Sheng et al., 2013). *Acidobacteria* responses to decreasing pH gradient uphill as observed in our current study (**Figure 2**) co-aligns with that of studies from diverse array of 87 soils across North and South America by Jones et al. (2009), where they combined pyrosequencing and clone library to arrive at the conclusion. The significant negative correlation of *Acidobacteria* abundance to TN in our study was also corroboratory to finding by Foesel et al. (2014). The influence of pH, however, in the grassland soils was found positively correlated to *Acidobacteria* abundance which is in contrast to our current finding and to that of Jones et al. (2009). Comparative genomics involving three genomes of *Acidobacteria* suggests their role in nitrogen cycling in soils and sediments is the reduction of nitrate, nitrite, and possibly nitric oxide (Ward et al., 2009). The soil microbial communities across vertical climate zones on the Tibetan Plateau also showed an elevational zonation feature, and distribution patterns of soil microbes were regulated mainly by soil pH, SOC and TN (Finkel et al., 2012). Within this phylum family of *Coriobacteriaceae, Solibacteraceae*, and *Acidobacteriaceae* were highly dominant with genus level classification of *Candidatus koribacter* and *C. solibacter.* A clone library approach (Shivaji et al., 2011) to understand the bacterial diversity in the Pindari glacier in the Himalayan mountain ranges India, showed 15.9% of the clones were represented by *Acidobacteria* in the library with genera identified as *Acidobacterium, Bryobacterium, Geothrix, Koribacter*, and *Solibacter*. Comparing three genome of *Acidobacteria* showed large class of high-molecular weight excreted proteins suggesting important factors for desiccation resistance, biofilm formation and soil structure modeling. Apart from presence of polyketide synthase and macrolide synthase, which shows high propensity of novel antimicrobial sources, exhibition of slow metabolic rates under low nutrient conditions equip them to tolerate fluctuations in soil hydration (Ward et al., 2009). Colonization of *Acidobacteria* in *Rhododendron* rhizosphere might shed light on adaption biology and plant–microbe interactions.

*Actinobacteria* occurred substantially at all four sites; however, the proportion in the rhizosphere was more at the upper region than lower. None of the measured parameters accounted for the changes in actinobacterial community differences, suggesting other factors not studied in the current study might account for the change. In a culture-independent survey of high elevation mineral soils from the dry valleys of Himalaya, the diversity of bacteria varied similar to the dry valleys of Antarctica (Costello et al., 2009). They reported the dominant groups in high altitude Himalayas were *Actinobacteria, Acidobacteria*, and *Cyanobacteria*, whereas in high Andes were found to be *Actinobacteria, Chloroflexi*. It is noteworthy that the *Chloroflexi* phylum does not represent most of its phylotypes in low-elevation habitats but inhabits cold and plant dominated high elevation ecosystems (Costello and Schmidt, 2006; Freeman et al., 2009). A previous detailed study in cool temperate mountain in Northwest of Yunnan, China at 3000–3900 m with annual temperature of 5°C reported *Streptomyces, Micromonospora, Nocardia*, and *Thermoactinomyces* strains where 97% constituted of *Streptomyces* genera. Also the snowy mountain in the same region at 3900–4500 which experiences average annual temperature below 0°C and represented by vegetation of *R. primulaeflorum*, *R. nivale* among others showed *Streptomyces* populations of 83% (Xu et al., 1996). Psychrophilic *Actinomycetes* genera identified were *Streptomyces*, *Micromonospora, Actinomadura, Saccharopolyspora*, and *Nocardia*. A recent study pertaining to the deglaciated high altitude soils of Himalaya along transacts from 5000 to 6000 m corresponded to equally likely phylogenetically distant and closely related communities at 50 m short distances and no significant difference in community structure was observed in response to time and season. *Acidobacteria, Proteobacteria, Verrucomicrobia*, and *Actinobacteria* were encountered at 21, 19.8, 14.9, and 10.3% average frequency, respectively (Stres et al., 2013).

It is interesting to notice the prevalence of *Rhodococcus*, an important genus of the family *Nocardiaceae*, phylum *Actinobacteria* with higher abundance in the lower altitudinal region. *Rhodococcus* is an endophytic bacterium which represents an enormous untapped source of microbial diversity and prospect of novel bioactive products. The wealth of *Rhodococcus* reported from North Eastern Asia suggests its importance. Example species include, *Rhodococcus kroppenstedtii* a novel species from Lahaul-Spiti Valley in Himalayan cold desert, India (Mayilraj et al., 2006), *Rhodococcus kunmingensis* from Taxus chinensis rhizosphere in Kunming, China (Wang et al., 2008), *Rhodococcus kyotonensis* from Kyoto, Japan (Li et al., 2007) and *Rhodococcus cercidiphylli*, an endophyte identified from leaf sample of *Cercidiphyllum japonicum* from Yunnan province, South-West China (Li et al., 2008). Moreover with increasing altitude, biological processes are posed with the challenge of increased irradiation by ultraviolet rays. Albarracín et al. (2013) reported isolated strains of *Rhodococcus* displaying an intrinsic and high UV-B resistive capability. These findings might suggest the selective colonization of specific *Rhodococcus* populations in the rhizosphere at the altitudinal sites of PTSO.

The dominant OTU’s were further explored at the species level for their closest validly published relative available in RDP or Silva by searching against EZtaxon-E One dominant OTU with cultured representative in RDP identified to be *B. valentinum* (JX514883) was also isolated from the rhizosphere of *L. mariae-josephi* endemic plant, and was equally abundant in both the lower altitude soil in our present study (**Table 2**). Navaro et al. (2014) have shown that specific inoculation of *Bradyrhizobium* symbiont strains in seeds of *L. mariae-josephi*, can be significant approach toward survival and recovery of an endangered legume species in its natural environment, and suggest it to be a significant approach toward conservation of endangered plant species. Interesting, plant specific root exudates viz; flavonoids, a diverse class of polyphenols secreted by soybean roots attracts the beneficial bacterium *B. japonicum* thereby serving as signals in plant microbe interaction (Morris et al., 1998). Moreover flavonoids isolated from white lupin roots additionally mobilized inorganic phosphorous and decreased soil microbial respiration, citrate mineralization and soil phosphohydrolase (Tomasi et al., 2008). Similarly, OTU_4 identified to be Rhodoplanes taxonomically was closest to unclassified Rhizobiales (AB240329) and was isolated from the biofilm of Phragmite rhizosphere. Although the structure of *Proteobacteria* sub-community was explained by TN content (Supplementary Figure S3), negative correlation between relative abundance and NO_3_^+^ was observed. High abundance of *Bradyrhizobium* and Rhodoplanes from sequence reads suggests their important role in N_2_ fixation in the rhizosphere (Yarwood et al., 2009). Other OTU’s having closest match to RDP sequences were uncultured clones or DGGE fragment isolated from diverse environments such as bamboo forest at high elevation, boreal forest soil with pH and Nitrogen gradient, un-vegetated soil at Singy Island, Antarctic habitat, snow melt soil at Alps.

The Indian Himalayan mountain ecosystem is one the most fragile in the world and the biodiversity of the region is vulnerable due to unprecedented stress owing to human perturbations. Our study concerns one of the medicinal plants endemic to the region and finding of this study will aid in maneuvering *in situ* as well as *ex situ* conservation strategies. We conclude by stating that, bacterial community differences in the rhizosphere of *R. arboretum* Sm. subsp. delavayi in the EH high altitude can be mostly predicted by pH, TN, OM. We believe our study is the first of its kind from this region summarizing the rhizospheric bacterial population of medicinally important endemic *R. arboretum* Sm. subsp. delavayi growing at high altitudes in cold climatic conditions. Although pH influence on bacterial community responses is a documented phenomenon, our study provides additional support on soil edaphic factors influence on different dominant taxas thriving at high altitudes experiencing extremes of weather conditions. The shift of acidobacterial relative abundance with pH changes provides evidence of niche specialization of acidobacterial subpopulations in high altitude cold soil. With rapid changes in biogeography and selection over time, the bacterial community is also undergoing a gradual replacement; hence findings of the current study will be invaluable assessors in understanding community ecology shifts and in long term sustainability, conservation of *R. arboreum.*

## Author Contributions

Conceived and designed the experiments: RD and RS. Performed the experiments: RD, AY, and RS. Bioinformatics and statistical data analysis: RD. Contributed reagents/materials/Analysis tools: RS and RD. Manuscript preparation: RD. Contributed in critically reviewing/correction: VG, BS, and PH.

## Conflict of Interest Statement

The authors declare that the research was conducted in the absence of any commercial or financial relationships that could be construed as a potential conflict of interest.
