# Supplementary material for: Rhizospheric Bacterial Community of Endemic Rhododendron arboreum Sm. Ssp. delavayi along Eastern Himalayan Slope in Tawang
Source: Front Plant Sci. 2016 Sep 2;7:1345. doi: 10.3389/fpls.2016.01345 (PMC5009118; doi:10.3389/fpls.2016.01345)
Supplement: Supplementary file 1 [file Data_Sheet_1.PDF]

Bacterial composition of endemic *Rhododendron arboreum* Sm. ssp. delavayi rhizosphere under cold climatic conditions along the Eastern Himalayan slope, Tawang, India

Rajal Debnath[1], Archana Yadav[1], Bhim Pratap Singh[2], Vijay K. Gupta[3], Ratul Saikia[1]*

[1]Microbial Biotechnology Laboratory, Biotechnology Division, CSIR-North East Institute of Science and Technology, Jorhat 785006, Assam, India

[2]Department of Biotechnology, Mizoram University, Aizwal, India.

[3]Molecular Glycobiotechnology Group, Discipline of Biochemistry, National University of Ireland Galway, Galway, Ireland.

*Email: rsaikia19@gmail.com

Table S1 Physico-chemical properties of the rhizosphere soil (RS) from the four different altitudinal sites.

SampleID	Temp	OM (%)	Soil_pH	TN (mg/kg)	TC (g/100g)	C:N	P(mg/100g)	Mg(mg/kg)	Moisture (%)	NO_3^+ (µg N g^{-1} soil)	NH_4^+ (µg N g^{-1} soil)
P.B.4	5.2	3.02 ± 0.05	4.76 ± 0.33	2142 ± 134.42	4.1 ± 1.2	18.14 ± 7.77	18.8 ± 2.54	307 ± 54.53	2.78 ± 0.54	1.49 ± 0.43	23.67 ± 1.43
P.C.8	5.3	2.07 ± 0.08	4.12 ± 0.16	2491 ± 112.6	2.5 ± 0.32	11.04 ± 2.84	17.75 ± 4.32	315.24 ± 43.56	1.5 ± 0.43	1.17 ± 0.76	23.03 ± 2.67
Y.9.1	6.3	3.04 ± 0.12	5.12 ± 0.13	723 ± 23.54	2.5 ± 0.43	34.60 ± 7.23	50 ± 9.54	337.6 ± 22.43	3.5 ± 1.24	1.67 ± 0.13	24.03 ± 2.87
N.D.1	5.8	3.17 ± 0.09	4.37 ± 0.54	2427 ± 53.67	3.6 ± 1.03	14.73 ± 9.14	18.34 ± 1.4	318.26 ± 42.34	5.7 ± 1.67	1.73 ± 0.87	24.14 ± 3.62
B.6.8	5.6	3.45 ± 0.32	5.61 ± 0.31	1286 ± 143.24	2.4 ± 0.54	18.58 ± 3.24	43.56 ± 4.87	342.43 ± 12.3	4.48 ± 0.78	1.92 ± 0.21	24.43 ± 4.32
N.4.8	4.9	2.22 ± 0.16	5.23 ± 0.2	1398 ± 121.2	0.84 ± 0.09	5.95 ± 0.74	18.23 ± 4.32	148.34 ± 2.45	2.04 ± 0.32	1.3 ± 0.08	23.28 ± 1.76
P.E.1	5.3	1.76 ± 0.03	3.99 ± 1.02	1367 ± 83.52	1.7 ± 0.75	12.37 ± 7.68	53.23 ± 8.54	325.1 ± 5.43	1.24 ± 0.22	1.11 ± 0.07	22.9 ± 2.08
N.1.4	4.8	2.12 ± 0.09	4.23 ± 0.76	1706 ± 43.25	1.3 ± 0.432	8.42 ± 9.99	18.34 ± 8.65	149.34 ± 31.21	1.72 ± 0.12	1.23 ± 0.21	23.14 ± 3.02
Y.3.8	3.2	3.61 ± 0.54	5.7 ± 1.06	966 ± 175.32	2.86 ± 1.54	29.88 ± 5.78	26.78 ± 7.64	275.78 ± 23.65	4.66 ± 0.29	1.96 ± 0.87	24.61 ± 6.04
Y.5.4	2.7	2.74 ± 0.32	4.89 ± 0.42	815 ± 162.3	3.1 ± 1.22	38.04 ± 6.52	25.2 ± 5.21	270.8 ± 18.43	3.04 ± 1.03	1.55 ± 0.42	23.8 ± 1.43
B.7.4	4.2	4.13 ± 0.13	6.2 ± 0.88	2029 ± 112.3	1.56 ± 0.69	7.53 ± 2.14	20 ± 6.43	138.2 ± 18.53	5.66 ± 0.42	1.98 ± 0.11	25.11 ± 6.05
B.10.1	2.8	3.63 ± 0.37	5.8 ± 1.14	1276 ± 143.2	2.56 ± 0.97	19.06 ± 4.77	24.89 ± 11.32	279.33 ± 32.88	4.86 ± 1.16	2.01 ± 1.07	25.71 ± 4.07

N, total nitrogen measured in mg/kg; C, total carbon measured in g/100g; Mg, magnesium measured in milligrams per kilogram; Moisture, percentage of moisture present within sample; NO_3^+, nitrate measured in microgram per gram; NH_4^+, ammonium measured in microgram per gram; OM, percentage of organic matter; P, phosphorus measured in milligrams per kilogram; pH, acidity hydrogen ion concentration; Temp, atmospheric temperature.

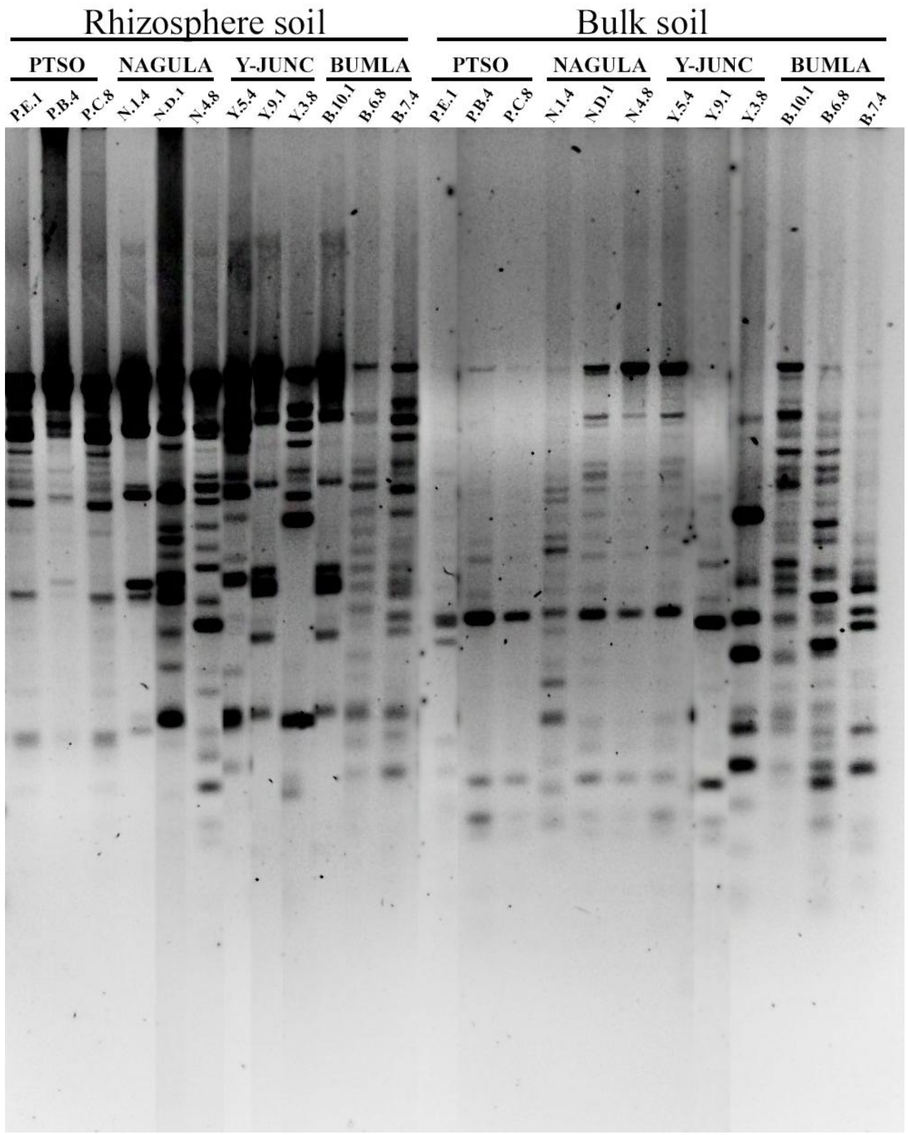

Fig S1| Bacteria DGGE fingerprint. Samples were taken from bulk soils and the corresponding R. arboretum rhizosphere at different altitudes PTSO, Nagula, Y-junction and Bum La.

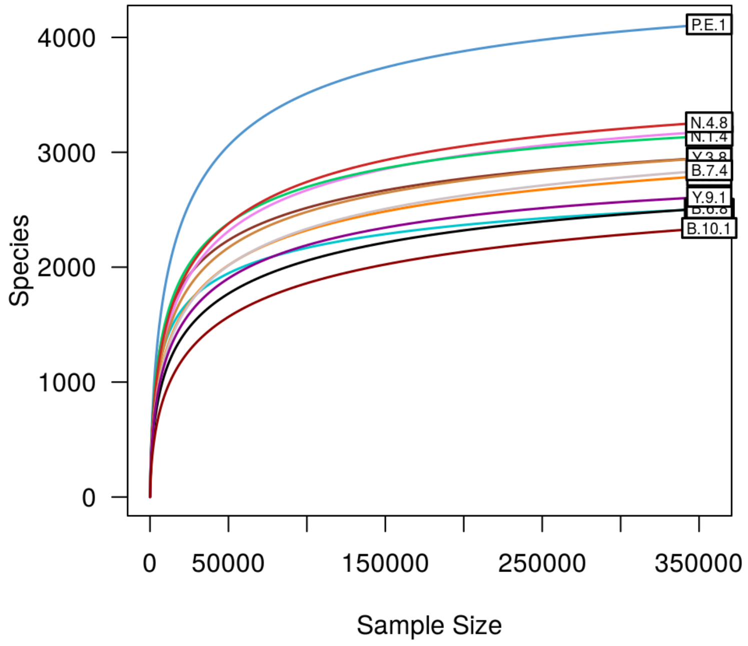

Fig. S2 Rarefaction curves of the 12 rhizospheric samples generated after rarefying at even sampling depth (min reads 358728).

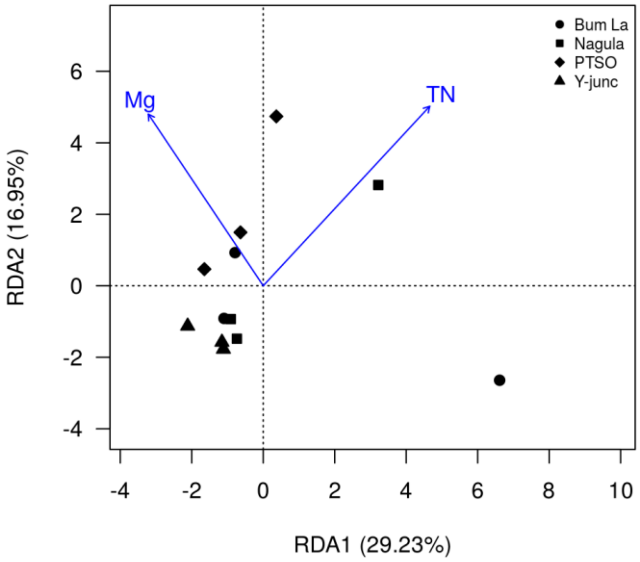

Fig. S3 PCoA of different soil parameters influencing the sub-communities of *Proteobacteria* phylum. Each axis explaining the percentage of the total variance is included in the parenthesis of axis label. Only

discriminative soil parameters significantly ($p<0.05$ by 1000 times permutation tests) explaining the community variation are shown.

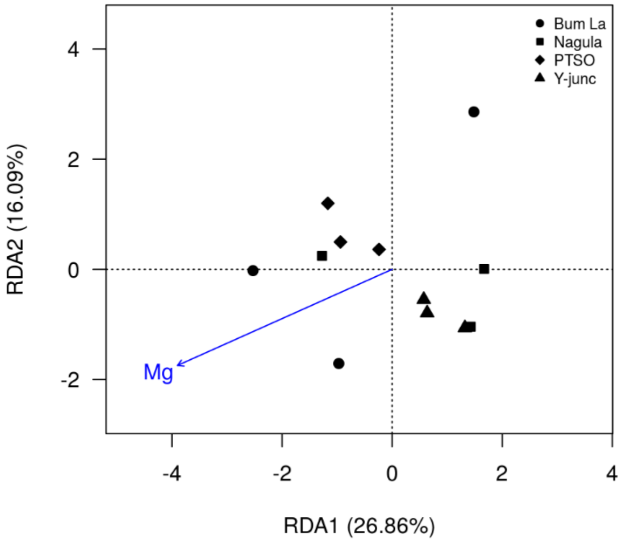

Fig. S4 PCoA of different soil parameters influencing sub-communities of *AD3* phylum. Each axis explaining the percentage of the total variance is included in the parenthesis of axis label. Only discriminative soil parameters significantly ($p<0.05$ by 1000 times permutation tests) explaining the community variation are shown.

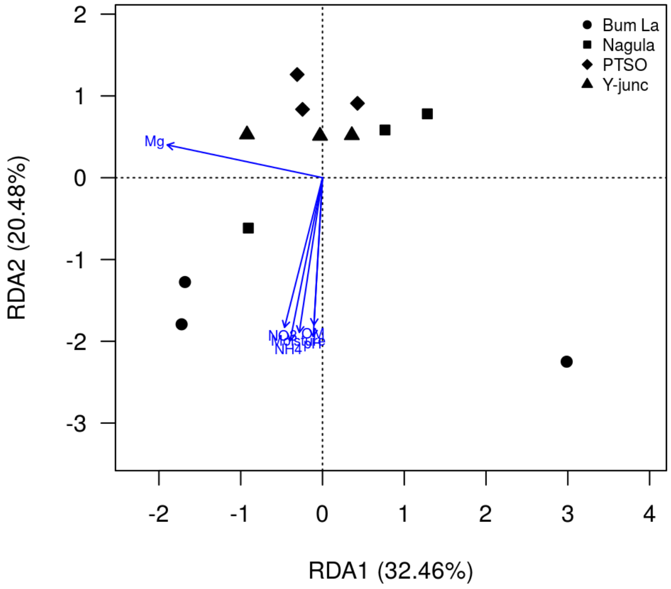

Fig. S5 PCoA of different soil parameters influencing the sub-communities of *WPS-2* phylum. Each axis explaining the percentage of the total variance is included in the parenthesis of axis label. Only discriminative soil parameters significantly (p<0.05 by 1000 times permutation tests) explaining the community variation are shown.

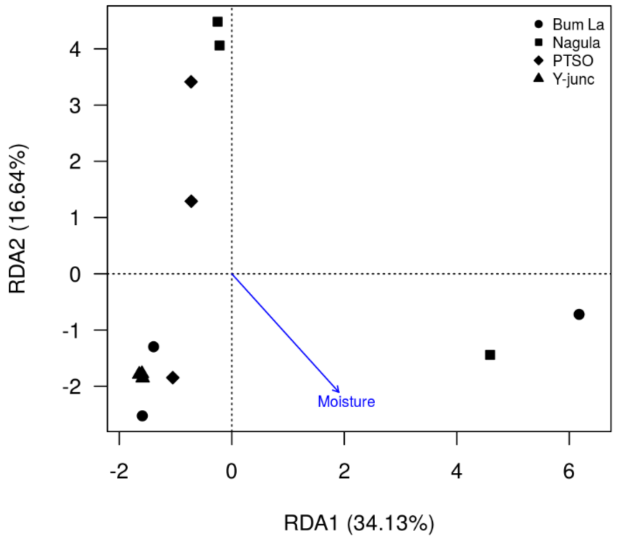

Fig. S6 PCoA of different soil parameters influencing sub-communities of *Chloroflexi* phylum. Each axis explaining the percentage of the total variance is included in the parenthesis of axis label. Only discriminative soil parameters significantly (p<0.05 by 1000 times permutation tests) explaining the community variation are shown.

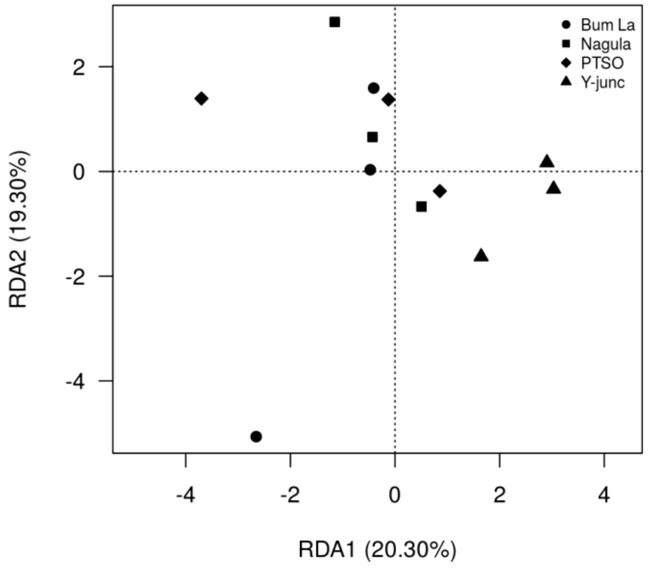

Fig. S7 PCoA of different soil parameters influencing sub-communities of *Firmicutes* phylum. Each axis explaining the percentage of the total variance is included in the parenthesis of axis label. Only discriminative soil parameters significantly ($p<0.05$ by 1000 times permutation tests) explaining the community variation are shown.

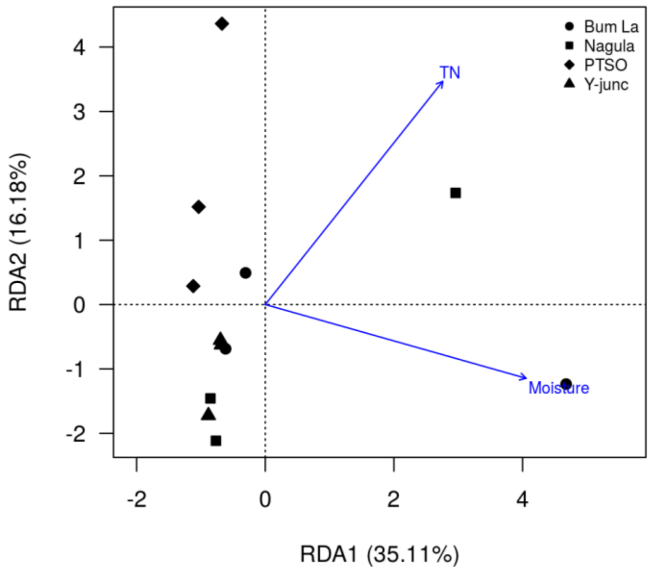

Fig. S8 PCoA of different soil parameters influencing sub-communities of *Gemmatimonadetes* phylum. Each axis explaining the percentage of the total variance is included in the parenthesis of axis label. Only discriminative soil parameters significantly ($p<0.05$ by 1000 times permutation tests) explaining the community variation are shown.

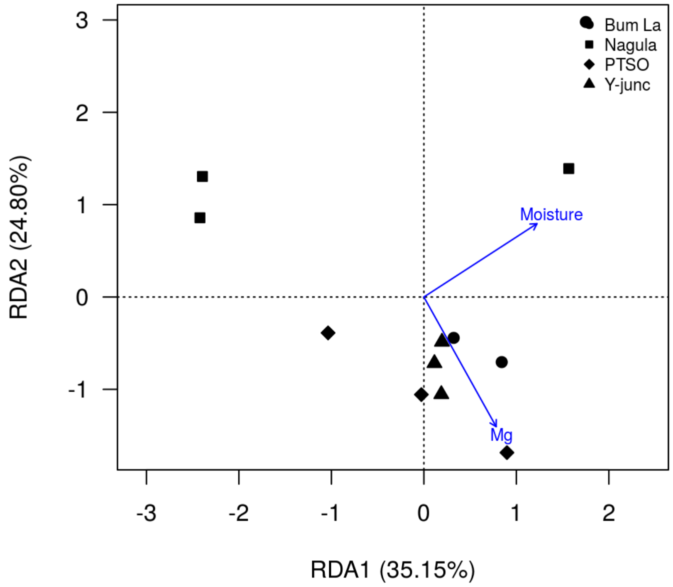

Fig. S9 PCoA of different soil parameters influencing sub-communities of *Nitrospirae* phylum. Each axis explaining the percentage of the total variance is included in the parenthesis of axis label. Only discriminative soil parameters significantly ($p<0.05$ by 1000 times permutation tests) explaining the community variation are shown.

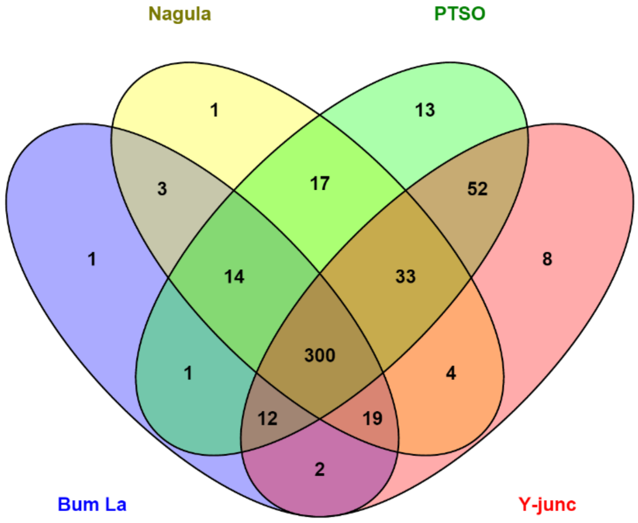

Fig. S10 Shared phylotypes among the four sites studies. The extent of overlap shows a total of 300 OTU's represented out of 490 OTU's clustered at 3% genetic distance and agglomerated at the level of species.
